# Choosing a hospital assistance ship to fight the covid-19 pandemic

**DOI:** 10.11606/s1518-8787.2020054002792

**Published:** 2020-08-05

**Authors:** Igor Pinheiro de Araújo Costa, Sérgio Mitihiro do Nascimento Maêda, Luiz Frederico Horácio de Souza de Barros Teixeira, Carlos Francisco Simões Gomes, Marcos dos Santos

**Affiliations:** I Universidade Federal Fluminense Departamento de Pós-Graduação em Engenharia de Produção NiteróiRJ Brasil Universidade Federal Fluminense (UFF). Departamento de Pós-Graduação em Engenharia de Produção. Niterói, RJ, Brasil; II Centro de Análises de Sistemas Navais Rio de JaneiroRJ Brasil Centro de Análises de Sistemas Navais (CASNAV). Rio de Janeiro, RJ, Brasil; III Instituto Militar de Engenharia Rio de JaneiroRJ Brasil Instituto Militar de Engenharia (IME). Rio de Janeiro, RJ, Brasil

**Keywords:** Public Health, Pandemics, Relief work, Coronavirus infection

## Abstract

**OBJECTIVE:**

To apply the THOR 2 multi-criteria support system to select the Brazilian navy's most suitable hospital care vessel (NAsH) to support the fight against the covid-19 pandemic.

**METHODS:**

We used the first three stages of the Soft Systems Methodology for structuring and modeling of the problem. For the evaluation and ordering of alternatives, we used the Thor 2 multi-criteria support system, comparing four classes of NAsH in the light of their operational and hospital criteria: “Dr. Montenegro,” “Soares Meirelles,” “Oswaldo Cruz” and “Tenente Maximiano.” The chosen ship would support the amazon hospital system, which has an increasing number of cases of covid-19.

**RESULTS:**

After the application of the methods, we analyzed three distinct scenarios of ordering the alternatives, which allowed a robust sensitivity analysis, conferring greater transparency and reliability to the decision-making process. The NAsH “Oswaldo Cruz” was selected to be used in the fight against the pandemic.

**CONCLUSIONS:**

This study brings valuable contribution to academia and society, since it represents the application of a multi-criteria decision-aid method in the state of the art to contribute to the solution of a real problem that affects millions of people in Brazil and worldwide.

## INTRODUCTION

The new coronavirus disease pandemic (covid-19) apparently represents the biggest and fastest challenge for public health systems in decades. As the virus and its impact spread, health systems around the world respond with large-scale protection measures and resource reallocation to contain its advance^1^.

The international dissemination of cases of covid-19 was rapid and intense due to the facilities of displacement between countries in modern life. This has led these countries authorities to adopt non-pharmaceutical control measures to avoid transmission, such as social isolation^2^.

According to Ozamiz-Etxebarria et al.^3^, the pandemic is bringing profound consequences to the global economy: millions of people lost their jobs and trillions of dollars evaporated from stock exchanges around the world before they closed their doors to prevent an absolute collapse, either because brokers got sick or because financial assets plummeted.

To reduce the damage associated with covid-19, urgent measures to control infection and public health are necessary to limit the global spread of the virus^4^. In resource-poor environments, countries have little time to prepare prevention and management strategies, including the identification of high-risk populations and regions^5^.

Studies and recommendations from experts have identified strategies to increase hospital capacity and manage the flow of patients, among which the naval service care stands out. The United States, for example, has used the USNS Comfort, a hospital ship with a capacity of 1,200 beds^6^. This vessel was built in 1976 as a San Clemente-class oil tanker and converted into a hospital ship in 1987. The USNS Comfort was deployed for humanitarian aid operations in New York, Haiti, New Orleans, the Persian Gulf, and Puerto Rico. Psychiatrists, psychologists, nurses and behavioral health technicians integrated these missions^7^.

According to the Brazilian Medical Association^8^, the health system in Manaus is collapsed since late April 2020 and has no prospect of improvement, given the increasing number of cases of covid-19 in the region. With the capacity of Brazilian hospitals increasingly close to exhaustion, the Armed Forces, especially the Brazilian Navy, emerge as allies of the Federal Government in fighting the pandemic, which puts a large part of the population at risk. The use of Brazilian Navy's hospital care ships (NAsH), subordinate to the Command of the 9th Naval District in Manaus, could reduce hospital demand and help fight the pandemic in the state of Amazonas.

A NAsH is a ship operated by the Brazilian Navy that can be specially designed, built or eventually adapted to the functions of floating hospital, with periodic passage through the special health centers. These centers are strategic places dedicated for the performance of the NAsH, ensuring medical and dental care, sanitary and health guidance, as well as epidemiological surveillance and fighting the endemic diseases of riverside populations^9^. The NAsH are named by the riverside populations as “ships of hope” due to its extremely important action, and because they are often the only alternative for medical care in these isolated regions^10^.

Our article analyzes four classes of the Brazilian Navy's NAsH; three of which operate in the Amazon Flotilla Command, subordinated to the Command of the 9th Naval District: “Oswaldo Cruz,” composed of two ships – “Oswaldo Cruz” (U18) and “Carlos Chagas” (U19) –; “Dr. Montenegro” (U16); and “Soares Meirelles” (U21). The fourth class – NAsH “Tenente Maximiano” – operates in the Command of the Flotilla of Mato Grosso, subordinated to the 6th Naval District Command (Ladário-MS). We analyze their operational and hospital capacities in the fight against covid-19 to employ them as hospital ships.

Given the limited number of beds in specialized units, it is not uncommon to consider early discharge or transfer to less complex units to provide vacancies for more severe cases^11^. Consequently, the NAsH would expand the availability of beds for treatment and recovery of patients, in addition to providing safety and timeliness for the intervention of specialists in complex cases. The selected ship would be used for the care of patients with non-contagious diseases, aiming to free hospital beds and favor the efforts of these hospital units to focus on fighting covid-19.

The installation of a hospital on board of a NAsH would support the health systems of several municipalities of Amazonas, from the capital Manaus to riverside regions, and even other states. We emphasize the possibility of using aircraft to support the operation of the ship, providing flexibility and speed in the transport of patients.

## METHODS

Production engineering becomes a fundamental mechanism in advising managers In the process of making the right decision^12^. Within this large area of engineering, operational research (OR) is the comprehensive and multidisciplinary field that employs mathematical and analytical models to solve complex problems of everyday life. The multi-criteria decision-aid method THOR 2 is the OR tool used in our article to select the most suitable ship. This tool will identify the alternative that best fulfills the floating hospital's mission in the support of the covid-19 pandemic.

One of the stages of the decision-making process includes problem structuring methods (PSM), which seek to organize themes and issues for which decision proposals are initially drawn up^13^. The PSM are widely accepted in the OR and in the movement of systems to understand and structure complex problems^14^, addressing situations with multipleactors, different perspectives, conflicting interests, significant intangible issues and complex uncertainties^15^.

Among the most commonly used methods, we chose the Soft Systems Methodology (SSM) for our study. Developed by Checkland^16^ and consolidated in literature^17^, SSM has been explored in different research fields, also serving equally diverse practical interests^13^. According to Checkland^16^, SSM presents seven stages of application, three of which will be used in our article to structure the problem: 1) explore an unstructured problematic situation; 2) express it; and 3) build brief definitions of relevant systems. In the first stage, the brainstorming technique was used to demonstrate the group's perceptions about all possible information, without interference or judgments to define the problem. In the second stage, a rich picture was built (Figure) to express all relevant aspects of the problem. The rich picture is a simple SSM tool, extremely useful for opening the discussion around individual perceptions towards a broad view of the different issues affecting the situation. They are created freely, not structured to capture the interpretation of participants of a real situation^16^^,^^17^.

**Figure:**
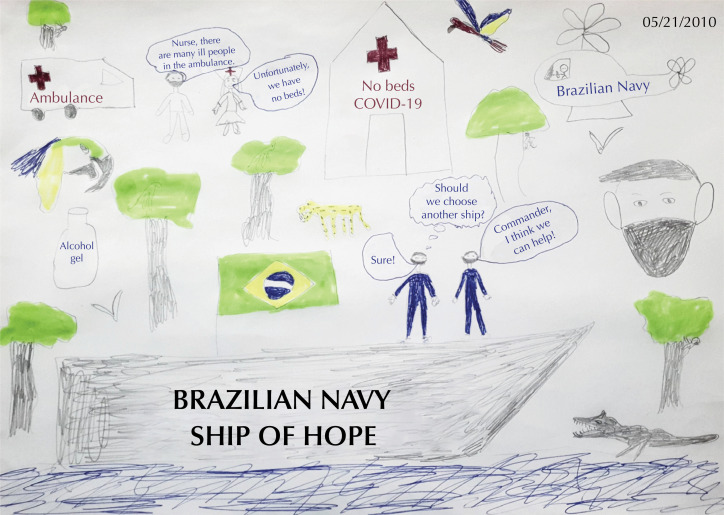
Rich picture, entitled “Navy's performance in Brazil in the fight against covid-19.”

In the third stage, after analysis of the rich picture, four relevant systems were identified: number of infected by the new coronavirus increasing on daily basis without signs of attenuation; collapse of the Amazon health system; possibility of assistance from the Brazilian Navy by the NAsH in the fight against the pandemic; and doubt about which NAsH would be more appropriate as floating hospital, considering that the use of all NAsH in the fight against the pandemic would hinder the provision of basic medical care in several riverside regions, since they often depends exclusively on the Navy hospital ships.

After applying the first three stages of SSM, we obtained the criteria that compose the problem. To fulfill the floating hospital mission, the ship must be able to provide assistance to the cities of Amazonas, from the capital Manaus to the riverside regions, that is, it must have great range of action, travel speed and autonomy so that it can provide unrestricted support, in the shortest time possible, to the regions far from its port headquarters, whose access can be difficult, also requiring good maneuverability of the vessel.

Regarding hospital capacity, the ship needs to be able to attend to non-contagious diseases, aiming to reduce the demand of the Amazon health system, which would focus only on fighting covid-19. The ship should have as few crew members as possible to have more space available to receive patients and accommodate them in beds that would compose a floating hospital. Moreover, the NAsH must have good capacity to evacuate patients to ground hospital units, either by speedboats or aircraft, providing speed and dynamism in patient care.

Based on the parameters presented in the analysis of the rich picture and after consulting Brazilian navy officers with more than 20 years of experience in the area, the following criteria were established:

- maximum speed: speed corresponding to the maximum power regime of the machines. The maximum speed of a warship is rarely used in peacetime, as it implies increased fuel consumption and unnecessary machine effort;
- radius of action: maximum distance, in nautical miles, that the ship can reach when leaving its base and returning to it without refuel;
- crew: set of hierarchically organized persons that ensure ship operation. A larger crew decreases the comfort and autonomy of the vessel when considering the supplies. In our study, we considered that larger crews are more disadvantageous;
- maneuverability: composition between the length and the ability of the ship to move, notably, in this case, when entering and docking in ports of smaller cities, with low depth and narrow space for maneuver, in addition to smaller piers;
- patient evacuation capacity: capacity of transferring patients, for medical reasons, to a health organization, or from the organization to the ship, either by smaller vessels (organic boats) or in aeromedical evacuation activities, extremely important for the efficient performance of a hospital ship, since they provide flexibility and speed in the transportation and care of patients; and
- hospital capacity: considers the types of medical care available, such as operating rooms and intensive care units (ICU), and the capacity of increasing the number of beds by installing a floating hospital.

Autonomy would be an extremely important criterion for the proposed analysis; however, since all ships evaluated have very similar values (in days), this criterion will not compose the problem.

The THOR method is based on three axiomatic concepts/theories for simultaneous use: preference modeling (approaching the French school – non-compensatory model), multi-attribute utility theory (bringing it closer to the American school – compensatory model) and theories that deal with inaccurate information. The combination of these theories allows quantifying the attractiveness of each alternative by creating a non-transitive aggregation function^18^. The use of THOR allows a faster and more efficient analysis of alternatives, considering the non-determinism of the weight assignment process, and quantifying this non-determinism, reapplying it in the process of ordering the alternatives^19^.

According to Gomes^20^, THOR main contributions to multi-criteria involve:

- a hybrid algorithm that encompasses concepts of the rough set theory (RST), fuzzy set theory (FST), utility theory and preference modeling;
- classification of discrete alternatives in transitive or non-transitive decision-making processes;
- elimination of redundant criteria, considering the possibility of duality in information using RST and inaccuracy in the decision-making process using FST.
- quantification of inaccuracy, using it in the multi-criteria decision-aid method;
- simultaneous entry of data from different decision makers, enabling them to express their value judgment(s) on a scale of reasons, intervals or ordinal;
- the possibility of decision-makers to work without assigning weights to such criteria if they are not able to, since they can use a resource that assigns weights to the criteria on an ordinal scale, which allows classifying THOR, therefore, as cardinal and partially ordinal method;
- elimination of the need to assign a value, usually arbitrary for agreement, according to some algorithms that have preference modeling as the basis.

To apply the methodology, the decision-maker must weigh the relative importance between the criteria, establish a limit of preference (p) and indifference (q) for each criterion (j), establish the disagreement and pertinence of the values of the weights attributed to each criterion, as well as the pertinence of the classification of the alternative in criterion^21^.

Given the inaccuracy and the lack of security in the judgment of the values used in the multi-criteria decision-aid methods, we think it is necessary to quantify the inaccuracy for each weight and each classification of alternatives. Decision-makers must express the levels of certainty through index of pertinence, associating a real number of the range with an element of the universe [0,1]. A pertinence index equal to 1 corresponds to absolute certainty, that is, the decision-maker is certain of the weight attributed to the criterion, whereas a pertinence index equal to 0 indicates absolute uncertainty. Two pertinence indices are used to reflect the degree of uncertainty of decision-makers, one referring to the weights of the criteria and the other to the classification of alternatives in each criterion^22^.

Given two alternatives, “a” and “b,” three situations should be considered when using THOR: S_1_, S_2_ e S_3_. When using the S_1_ algorithm, the alternatives only have their attractiveness punctuated when aP_j_b occurs. Index j represents each criterion analyzed. Thus, comparing the alternative “a” with the “b,” we identify the criteria in which aP_j_b occurs, considering the preference limits (P designates strict preference, Q designates weak preference), indifference (I designates indifference) and disagreement, verifying if the imposed condition is met. If satisfied, it is known that “a” dominates “b.” The ratios P, I and Q are expressed in equations 1, 2 and 3, respectively^23^:

(1) aPb↔g(a)−g(b)>+p

(2) aIb↔−q≤|g(a)−g(b)|≤+ q

(3) aQb↔q<|g(a)−g(b)|≤+ p

The notation “g(a)” represents the performance (value) of the alternative “a” in a given criterion.

Equations 4, 5 and 6 reflect the three situations so that one alternative is classified better than the other^23^:

(4) $$S1:\sum_{j=1}^{n}(w_{j}|aP_{j}b)>\sum_{j=1}^{n}\left(w_{j}|aQ_{j}b+aI_{j}b+aR_{j}b+bQ_{j}a+bP_{j}a\right)$$

(5) $$S2:\sum_{j=1}^{n}\left(w_{j}|aP_{j}b+aQ_{j}b\right)>\sum_{j=1}^{n}\left(w_{j}|aI_{j}b+aR_{j}b+bQ_{j}a+bP_{j}a\right)$$

(6) $$S3:\sum_{j=1}^{n}(w_{j}|aP_{j}b+aQ_{j}b+aI_{j}b)>\sum_{j=1}^{n}\left(w_{j}|aR_{j}b+bQ_{j}a+bP_{j}a\right)$$

Using the S_2_ algorithm, the alternatives only have their attractiveness punctuated when both aP_j_b and aQ_j_b occurs. Using the S_3_ algorithm, the alternatives only have their attractiveness punctuated when aP_j_b, aQ_j_b and aI_j_b occurs. In the algorithms S_2_ and S_3_, we see a more flexible scenario, in which a smaller difference between the alternatives allows classifying an alternative as better than the other^24^.

THOR is recommended preferably in situations of pseudocriteria and quasi-criterion since the method can be used at its full capacity. The use of THOR in situations of true criterion, when the values of p and q assume a value equal to zero, leads to the equality of the orders corresponding to S_1_ and S_2_^20^^,^^22^.

Among its main contributions, we emphasize the application of the THOR multi-criteria system in waste recycling in Brazil^20^ and in processes associated with health^22^. Gomes and Costa^18^ applied this method, together with the METHODS ELECTRE (I and II) and PROMETHÉE II, to the problem of choosing electronic payment models by credit card, and other authors used it to establish strategies for the purchase of a frigate opportunity for the Brazilian Navy^25^.

THOR only considers the multiplication by the index in the aQ_j_b situation, deteriorating the gain only in this case. THOR 2 is an evolution of the original THOR method, which also includes punctuation depreciation in situations of strong preference and indifference. Thus, THOR 2 represents a significant contribution since it quantifies all the uncertainty present in the attribution of the classifications of alternatives and weights.

To support our study, we conducted a literature research to obtain the operational and hospital data of each vessel. Many data are confidential, since NAsH are military ships; therefore, we used only the parameters available in official sources of the Brazilian Navy^26^^–^^29^.

Data were also collected in academic articles that address the performance of NAsH in civic-social actions^9^^–^^11^. This research originated the following data (Table 1).

**Table 1:** Operational and hospital data of the vessels evaluated.

Criterion	Characteristic	NAsH Dr. Montenegro	NAsH Oswaldo Cruz	NAsH Soares Meirelles	NAsH Tenente Maximiano
	Length (m)	42	47.2	63	31.06
	Width	11	8.45	12	6.5
Maneuverability	Draft	2.4	1.75	2.1	1.02
	Full load displacement (ton)	347	490	1,338	160
Crew	In number of people	60	27	47	23
Radius of action	In nautical miles	3,200 (5 knots)	3,000 (7 knots)	6,000(11 knots)	1,100(11 knots)
Maximum speed (riverdown)	In knots	10	12	12	12
Patient evacuation capacity	Patient evacuation features	2 speedboats for personnel transport	Flight deck able to operate a Bell helicopter Jet Ranger IH-6 or Esquilo UH-1 2, plus 2 speedboats for personnel transport	2 speedboats for personnel transport	2 speedboats for personnel transport
	Number of hospital beds available	6	6	6	3
Hospital capacity	Medical care available	3 offices, 2 dental offices, 1 laboratory, 1 pharmacy, 1 X-ray room, 2 hospital wards, 1 operating room, 1 emergency room, ICU	2 outpatient clinics, 2 dental offices, 1 laboratory, 1 pharmacy, 1 X-ray room, 2 hospital wards, 1 operating room	Offices, dental offices, pharmacy, vaccination room, X-ray room, operating room, hospital ward, clinical analysis laboratory	Surgical center, infirmary, sterilization room, purge room, pharmacy, laboratory, doctor's office, dental offices, 1 compartment equipped with X-ray device

## RESULTS

Table 2 shows alternatives, criteria, preference thresholds, disagreement and weights of the criteria used in the analysis. The first column consists of the alternatives, and the following six columns, of the criteria. Each cell corresponds to a ship alternative classified at its respective discretion. The alternatives of the maneuverability, hospital capacity and patient evacuation criteria were classified using a scale of intervals, in which we considered the relative difference among the values of the alternatives.

**Table 2:** Alternatives, criteria, weights and thresholds of preference and disagreement.

Ships	Maximum speed	Crew	Radius of action	Maneuverability	Hospital capacity	Patient evacuation
NAsH “Oswaldo Cruz”	10	-2 7	3,000	3	2	4
NAsH “Dr. Montenegro”	12	-60	3,200	3	2	2
NAsH “Soares Meirelles”	12	-47	6,000	1	3	2
NAsH “Tenente Maximiano”	12	-23	1,100	4	1	1

	Criteria	Weight assigned	P value	Q value	Disagreement	
	Maximum speed	3	2	1	4.5	
	Crew	1	11	5	18	
	Radius of action	2	1,000	300	2,500	
	Maneuverability	2	1	0.5	3.1	
	Hospital capacity	4	1	0.5	2.9	
	Patient evacuation	4	1	0.5	3.7	

The values of weights, preference limits (p), limits of indifference (q) and disagreement for each criterion were attributed by joint analysis with specialists in the area.

Using the data from Table 2, we could generate the orderings listed in Table 3. The calculations were performed using a computational system called THOR 2, developed by Tenório et al.^30^ at the Instituto Militar de Engenharia (IME – Military Engineering Institute).

**Table 3:** Results obtained after the application of the method.

S_1_		S_2_		S_3_	
NAsH “Tenente Maximiano”	1.5	NAsH “Oswaldo Cruz”	1.881	NAsH “Oswaldo Cruz”	2.357
NAsH “Oswaldo Cruz”	1.5	NAsH “Soares Meirelles”	1.714	NAsH “Soares Meirelles”	1.857
NAsH “Dr. Montenegro”	1.5	NAsH “Tenente Maximiano”	1	NAsH “Tenente Maximiano”	1
NAsH “Soares Meirelles”	1.5	NAsH “Dr. Montenegro”	0.5	NAsH “Dr. Montenegro”	0.5

## DISCUSSION

In situation S_1_, the four ships obtained exactly the same score. Therefore, we could not conclude which ship would be the best to the mission.

In algorithm S_2_, which considers the overclassification ratios aP_j_b and aQ_j_b to dominance, the NAsH “Oswaldo Cruz” had a slightly higher sum than “Soares Meirelles,” being mathematically the best ship in the proposed analysis. However, in practice, the two can be considered technically even due to the extremely small relative difference between them (0.167). Also in scenario S_2_, it is observed that the NAsH “Tenente Maximiano” and “Dr. Montenegro” presented the lowest scores, with higher relative differences when compared with the two best classified ships and can be discarded from the selection process.

Regarding S_3_ algorithm, the most flexible situation, the NAsH “Oswaldo Cruz” obtained again the highest score, but with a more considerable relative difference to the second place, which confirms its choice as the most appropriate ship to be used in the fight against the pandemic.

Therefore, we obtained the following final classification: first, NAsH “Oswaldo Cruz; second, NAsH “Soares Meirelles”; third, NAsH “Tenente Maximiano”; and fourth, NAsH “Dr. Montenegro.”

Evaluating the reasons that led to the final classification of the alternatives, we observe that “Oswaldo Cruz” and “Soares Meirelles” obtained the two best results due to the highest evacuation and hospital capacities, respectively – the two criteria with greater weight (4) assigned by the specialists in the proposed analysis. The “Soares Meirelles” obtained the highest score in three of the six criteria evaluated; however, it did not obtain good grades in the other criteria. The “Oswaldo Cruz,” in turn, obtains the highest score only in the evacuation capacity, maintaining regularity in the other criteria, thus becoming the most indicated ship. The capacity of operating with aircraft may have been the differential factor for choosing the NAsH “Oswaldo Cruz,” making it obtain the highest note of evacuation capacity and with greater relative distance.

Therefore, we have clearly achieved our objective by pointing out the NAsH “Oswaldo Cruz” as the most appropriate ship for fighting the pandemic in the Amazon. THOR 2 method can notably be used to solve real problems of the most varied types – tactical, operational and strategic – thus being a very useful method for decision making.

Moreover, the ease, flexibility, reliability and speed of application of the method can greatly facilitate the often complicated calculations that involve multi-criteria decision aid.

Finally, we suggest this model of ordering alternatives using THOR 2 to be further applied in the health area.
