# Supplementary material for: Choosing a hospital assistance ship to fight the covid-19 pandemic
Source: Rev Saude Publica. 2020 Aug 5;54:79. doi: 10.11606/s1518-8787.2020054002792 (PMC7416598; doi:10.11606/s1518-8787.2020054002792)
Supplement: Translation

# Translation: Escolha de navio de assistência hospitalar no combate à pandemia da covid-19

**Authors:** Igor Pinheiro de Araújo Costa, Sérgio Mitihiro do Nascimento Maêda, Luiz Frederico Horácio de Souza de Barros Teixeira, Carlos Francisco Simões Gomes, Marcos dos Santos

**Affiliations:** I NiteróiRJ Brasil Universidade Federal Fluminense (UFF). Departamento de Pós-Graduação em Engenharia de Produção. Niterói, RJ, Brasil; II Rio de JaneiroRJ Brasil Centro de Análises de Sistemas Navais (CASNAV). Rio de Janeiro, RJ, Brasil; III Rio de JaneiroRJ Brasil Instituto Militar de Engenharia (IME). Rio de Janeiro, RJ, Brasil

**Keywords:** Saúde Pública, Pandemias, Socorro em desastres, Infecções por Coronavírus

## Abstract

**OBJETIVO:**

Aplicar o método multicritério THOR 2 para selecionar o navio de assistência hospitalar (NAsH) da Marinha do Brasil mais indicado para apoiar o combate à pandemia de covid-19.

**MÉTODOS:**

Para a estruturação e modelagem do problema, foram usados os três primeiros estágios da *Soft Systems Methodology*. Já para a avaliação e ordenação das alternativas, foi utilizado o método de análise multicritério Thor 2, comparando quatro classes de NAsH à luz de seus critérios operativos e hospitalares: “Dr. Montenegro”, “Soares Meirelles”, “Oswaldo Cruz” e “Tenente Maximiano”. O navio escolhido apoiaria o sistema hospitalar do Amazonas, que apresenta número cada vez maior de casos de covid-19.

**RESULTADOS:**

Após a aplicação dos métodos, foi possível analisar três cenários distintos de ordenação das alternativas, o que permitiu uma análise de sensibilidade robusta, conferindo maior transparência e confiabilidade ao processo decisório. O NAsH “Oswaldo Cruz” foi selecionado para ser empregado no combate à pandemia.

**CONCLUSÕES:**

Este trabalho traz valiosa contribuição para academia e sociedade, uma vez que representa a aplicação de um método de auxílio à decisão multicritério no estado da arte para contribuir com a solução de um problema real que afeta milhões de pessoas no Brasil e no mundo.

## INTRODUÇÃO

A nova pandemia de doença do coronavírus (covid-19) aparentemente representa o maior e mais rápido desafio para os sistemas de saúde pública em décadas. À medida que o vírus e seu impacto se espalham, os sistemas de saúde em todo o mundo respondem com medidas de proteção em grande escala e realocação de recursos para tentar conter seu avanço^1^.

Devido às facilidades de deslocamento entre países na vida moderna, a disseminação internacional de casos de covid-19 foi rápida e intensa. Isso tem levado as autoridades a adotar medidas de controle não medicamentoso de transmissão, como isolamento social^2^.

De acordo com Ozamiz-Etxebarria et al.^3^, a pandemia está trazendo consequências profundas à economia global: milhões de pessoas perderam seus empregos e trilhões de dólares evaporaram das bolsas de valores por todo o mundo antes que fechassem suas portas para evitar um colapso absoluto, seja porque os corretores ficaram doentes ou porque os ativos financeiros despencaram.

Para diminuir os danos associados à covid-19 são necessárias medidas urgentes de controle de infecção e saúde pública para limitar a propagação global do vírus^4^. Em ambientes com poucos recursos, os países têm pouco tempo para preparar estratégias de prevenção e gestão, incluindo a identificação de populações e regiões de alto risco^5^.

Pesquisas e recomendações de especialistas identificaram estratégias para aumentar a capacidade hospitalar e gestão do fluxo de pacientes, dentre as quais se destaca o atendimento naval de pacientes. Os Estados Unidos, por exemplo, têm usado o USNS Comfort, navio-hospital com capacidade para 1.200 leitos^6^. Essa embarcação foi construída em 1976 como navio petroleiro da classe San Clemente e convertida em navio-hospital em 1987. O Comfort foi destacado para operações de ajuda humanitária em Nova York, Haiti, Nova Orleans, golfo Pérsico e Porto Rico. Psiquiatras, psicólogos, enfermeiros e técnicos de saúde comportamental integraram essas missões^7^.

De acordo com a Associação Médica Brasileira^8^, o sistema de saúde em Manaus está em colapso desde o final de abril de 2020 e não apresenta perspectiva de melhora, dado o crescente número de casos de covid-19 na região. Com a capacidade dos hospitais brasileiros cada vez mais próxima do esgotamento, as Forças Armadas, e em especial a Marinha do Brasil, surgem como aliadas do Governo Federal no combate à pandemia, que coloca em risco grande parte da população. Uma ação da Marinha que poderia desonerar hospitais e ajudar no combate à pandemia no estado do Amazonas seria a utilização de Hospitais de Campanha (HCamp) nos navios de Assistência Hospitalar (NAsH) subordinados ao Comando do 9° Distrito Naval, situado em Manaus.

Um NAsH é um tipo de navio operado pela Marinha brasileira que pode ser especialmente projetado, construído ou eventualmente adaptado para as funções de hospital flutuante, com passagem periódica pelos denominados polos de saúde. Estes polos são locais estratégicos para a atuação dos NAsH, assegurando assistência médica e odontológica e orientação sanitária e de saúde, além de vigilância epidemiológica e combate às endemias das populações ribeirinhas^9^. Graças a essa atuação extremamente importante, e por serem muitas vezes a única alternativa de atendimento médico nessas regiões isoladas, os NAsH são carinhosamente apelidados pelos ribeirinhos como “navios da esperança”^10^.

Neste artigo são analisadas quatro classes de NAsH da Marinha do Brasil, das quais três operam no Comando da Flotilha do Amazonas, subordinada ao Comando do 9° Distrito Naval: “Oswaldo Cruz”, composta por dois navios – “Oswaldo Cruz” (U18) e “Carlos Chagas” (U19) –; “Dr. Montenegro” (U16); e “Soares Meirelles” (U21). A quarta classe – NAsH “Tenente Maximiano” – opera no Comando da Flotilha de Mato Grosso, subordinada ao Comando do 6° Distrito Naval (Ladário-MS). Propõe-se analisar suas capacidades operativas e hospitalares no combate à covid-19 visando empregá-los como navios-hospitais.

Dado o limitado número de leitos em unidades especializadas, não é raro que se considerem alta precoce ou transferência para unidades menos complexas a fim de disponibilizar vagas para casos mais graves^11^. Com isso, além de proporcionar segurança e tempestividade para intervenção de especialistas em casos complexos, o NAsH ampliaria a disponibilidade de leitos para tratamento e recuperação de pacientes. O navio selecionado seria empregado nos atendimentos a pacientes de doenças não contagiosas, visando desonerar os leitos dos hospitais e possibilitar que os esforços dessas unidades hospitalares se concentrem no combate à covid-19.

A instalação de um HCamp a bordo de um NAsH possibilitaria apoiar os sistemas de saúde de diversos municípios do Amazonas, desde a capital Manaus até regiões ribeirinhas, e de outros estados. Outro ponto importante a ressaltar é a possibilidade de utilizar aeronaves no apoio à operação do navio, fornecendo flexibilidade e celeridade no transporte de pacientes.

## MÉTODOS

No processo para tomar a decisão correta, a engenharia de produção se torna mecanismo fundamental no assessoramento dos gestores^12^. Dentro desta grande área da engenharia, a pesquisa operacional (PO) é o campo abrangente e multidisciplinar que emprega modelos matemáticos e analíticos para solucionar problemas complexos do cotidiano. A ferramenta de PO usada neste artigo para selecionar o navio mais indicado para o cumprimento da missão será o método multicritério de apoio à decisão THOR 2, devendo ser identificada a alternativa que melhor cumpra a missão de HCamp no apoio ao combate à pandemia de covid-19.

Uma das etapas do processo de tomada de decisão compreende os métodos de estruturação de problemas (PSM, do inglês *Problem Structuring Methods*), que buscam organizar temas e perguntas para os quais são inicialmente elaboradas propostas de decisão^13^. Os PSM são amplamente aceitos na PO e no movimento de sistemas para entender e estruturar problemas complexos^14^, abordando, de forma geral, situações com múltiplos atores, diferentes perspectivas, interesses conflitantes, intangíveis significativos e incertezas complexas^15^.

Dentre os métodos mais comumente utilizados, neste artigo será utilizada a *Soft Systems Methodology* (SSM). Desenvolvida por Checkland^16^ e consolidada na literatura^17^, a SSM tem sido explorada em uma variedade de campos de pesquisa, servindo ainda a interesses práticos igualmente diversos^13^. De acordo com Checkland^16^, a SSM apresenta sete estágios de aplicação, dos quais três serão utilizados neste artigo para estruturar o problema: 1) explorar uma situação problemática não estruturada; 2) expressá-la; e 3) construir definições sucintas de sistemas relevantes. No primeiro estágio foi utilizada a técnica de *brainstorming* para demonstrar as percepções do grupo sobre todas as informações possíveis, sem interferências ou julgamentos para definir o problema. No segundo estágio foi construída uma figura rica (Figura), a fim de expressar todos os aspectos relevantes do problema. A figura rica é uma simples ferramenta de SSM, extremamente útil para abrir a discussão em torno das percepções individuais em direção a uma visão ampla sobre as diferentes questões que afetam a situação. São criadas de forma livre e não estruturada para capturar a interpretação dos participantes de uma situação real^16^^,^^17^.

**Figura:** Figura rica, intitulada “Atuação da Marinha no Brasil no combate à covid-19”.

No terceiro estágio, após análise da figura rica, foram identificados quatro sistemas relevantes: número de infectados pelo novo coronavírus crescendo a cada dia, sem sinais de atenuação; colapso do sistema de saúde do Amazonas; possibilidade de auxílio da Marinha do Brasil por meio dos NAsH no combate à pandemia; e dúvida sobre qual NAsH seria mais adequado como HCamp, tendo em vista que a utilização de todos os NAsH no combate à pandemia inviabilizaria o atendimento médico básico de diversas regiões ribeirinhas, cuja assistência em saúde muitas vezes depende exclusivamente da Marinha.

Após aplicar os três primeiros estágios da SSM, chega-se aos critérios que compõem o problema. Para cumprir a missão de HCamp, o navio escolhido deve ser capaz de prestar socorro às cidades do Amazonas, desde a capital Manaus até as regiões ribeirinhas, significando que deve ter grande raio de ação, velocidade de deslocamento e autonomia para que possa prestar apoio irrestrito, no menor tempo possível, às regiões distantes de seu porto-sede, cujo acesso pode ser difícil, exigindo também boa manobrabilidade da embarcação.

No que concerne à capacidade hospitalar, o navio precisa ter capacidade de atender a doenças não contagiosas, visando desonerar o sistema de saúde do Amazonas, o qual se concentraria apenas no combate à covid-19. Deve ter o menor número possível de tripulantes, visando ter mais espaço disponível para receber pacientes e acomodá-los em leitos que comporiam um HCamp a bordo. Além disso, é imprescindível que o NAsH possua boa capacidade de evacuação de pacientes para unidades hospitalares de terra, seja por meio de lanchas ou aeronaves, o que fornece celeridade e dinamismo no atendimento aos pacientes.

A partir dos parâmetros apresentados na análise da figura rica e após consulta a oficiais da Marinha do Brasil com mais de 20 anos de experiência na área, os seguintes critérios foram estabelecidos:

- velocidade máxima: velocidade correspondente ao regime de máxima potência das máquinas. A velocidade máxima de um navio de guerra é raramente utilizada em tempos de paz, pois implica maior consumo de combustível e esforço desnecessário da máquina;
- raio de ação: distância máxima, em milhas náuticas, que o navio consegue alcançar saindo de sua base e a ela regressando sem necessidade de reabastecer o combustível;
- tripulação: conjunto de pessoas hierarquicamente organizadas que asseguram a operação do navio. Uma tripulação mais numerosa diminui o conforto e a autonomia da embarcação no quesito mantimentos. Considerou-se, neste estudo, que tripulações maiores são mais desvantajosas;
- manobrabilidade: composição entre o comprimento e a capacidade do navio de se deslocar, notadamente, neste caso, ao adentrar e atracar em portos de cidades menores, com baixa profundidade e espaço estreito para manobra, além de cais e píer menores;
- capacidade de evacuação de pacientes: capacidade de transferir pacientes, por razões de ordem médica, para uma organização de saúde, ou desta para o navio, seja por embarcações menores (lanchas orgânicas) ou em atividades de evacuação aeromédica, extremamente importantes para a atuação eficiente de um navio-hospital, pois proporcionam flexibilidade e celeridade no transporte e atendimento de pacientes; e
- capacidade hospitalar: considera os tipos de atendimento médico disponíveis, como salas de cirurgia e unidades de terapia intensiva (UTI), e a capacidade de ampliar o número de leitos por meio da instalação de um HCamp a bordo.

Para a análise proposta, um critério extremamente importante seria a autonomia, mas como todos os navios avaliados apresentam valores (em dias) muito semelhantes, esse critério não comporá o problema.

O método THOR baseia-se em três conceitos axiomáticos/teorias para uso simultâneo: modelagem de preferência (aproximando-o da escola francesa – modelo não compensatório), teoria da utilidade multiatributo (aproximando-o da escola americana – modelo compensatório) e teorias que tratam da informação imprecisa. A utilização conjunta destas teorias permite quantificar a atratividade de cada alternativa ao criar uma função de agregação não transitiva^18^. O uso do THOR permite analisar as alternativas mais rápida e eficientemente, considerando o não determinismo do processo de atribuição de pesos, e quantificar esse não determinismo, reaplicando-o no processo de ordenação das alternativas^19^.

Segundo Gomes^20^, as principais contribuições do THOR ao multicritério envolvem:

- apresentar um algoritmo híbrido que engloba conceitos da teoria dos conjuntos aproximativos (TCA), teoria dos conjuntos nebulosos (TCN), teoria da utilidade e modelagem de preferências;
- ordenar alternativas discretas em processos decisórios transitivos ou não;
- eliminar critérios redundantes, levando em conta se há dualidade na informação por meio da TCA e se ocorre imprecisão no processo decisório mediante a utilização da TCN.
- quantificar a imprecisão, utilizando-a no método de apoio multicritério à decisão;
- permitir a entrada de dados simultâneos de diferentes decisores, possibilitando que expressem seu(s) juízo(s) de valor em escala de razões, intervalos ou ordinal;
- viabilizar que os decisores, caso não sejam capazes de atribuir pesos a tais critérios, trabalhem sem fazê-lo, uma vez que podem usar um recurso que atribui pesos aos critérios em escala ordinal, o que permite classificar o THOR, portanto, como método cardinal e parcialmente ordinal;
- eliminar a necessidade de atribuição de um valor, normalmente arbitrário para a concordância, conforme alguns algoritmos que têm a modelagem de preferências como base.
- Para aplicar a metodologia é necessário que o decisor represente a importância relativa entre os critérios na forma de um peso, estabeleça um limite de preferência (p) e de indiferença (q) para cada critério j, estabeleça a discordância e a pertinência dos valores dos pesos atribuídos a cada critério, assim como a pertinência da classificação da alternativa no critério^21^.

Diante da falta de segurança e da imprecisão no julgamento de valor empregado nos métodos de apoio multicritério à decisão, faz-se necessário quantificar a imprecisão para cada peso e para cada classificação das alternativas. Os decisores devem expressar os níveis de certeza por meio de índices de pertinência, associando a um elemento do universo um número real do intervalo [0,1]. Um índice de pertinência igual a 1 corresponde a absoluta certeza, ou seja, o decisor está plenamente seguro do peso atribuído ao critério, enquanto um índice de pertinência igual a 0 indica absoluta incerteza. Utilizam-se dois índices de pertinência para refletir o grau de incerteza dos tomadores de decisão, um deles referindo-se aos pesos dos critérios e o outro à classificação das alternativas em cada critério^22^.

Dadas duas alternativas, “a” e “b”, três situações devem ser consideradas na utilização do THOR: S_1_, S_2_ e S_3_. Na utilização do algoritmo S_1_, as alternativas só têm sua atratividade pontuada em situações nas quais ocorre aP_j_b. O índice j representa cada critério analisado. Desta forma, comparando a alternativa “a” com a “b”, identificam-se os critérios em que ocorre aP_j_b, levando em consideração os limites de preferência (P designa preferência estrita, Q designa preferência fraca), indiferença (I designa indiferença) e discordância, verificando se a condição imposta é satisfeita. Se for satisfeita, sabe-se que “a” domina “b”. As relações P, I e Q estão expressas nas equações 1, 2 e 3, respectivamente^23^:

(1) aPb↔g(a)−g(b)>+p

(2) aIb↔−q≤|g(a)−g(b)|≤+q

(3) aQb↔q< |g(a)−g(b)| ≤+p

A notação “g(a)” representa o desempenho (valor) da alternativa “a” em um determinado critério.

As equações 4, 5 e 6 refletem as três situações para que uma alternativa seja classificada melhor do que a outra^23^:

(4) $$S1:\sum_{j=1}^{n}(w_{j}|aP_{j}b)>\sum_{j=1}^{n}\left(w_{j}|aQ_{j}b+aI_{j}b+aR_{j}b+bQ_{j}a+bP_{j}a\right)$$

(5) $$S2:\sum_{j=1}^{n}\left(w_{j}|aP_{j}b+aQ_{j}b\right)>\sum_{j=1}^{n}\left(w_{j}|aI_{j}b+aR_{j}b+bQ_{j}a+bP_{j}a\right)$$

(6) $$S3:\sum_{j=1}^{n}(w_{j}|aP_{j}b+aQ_{j}b+aI_{j}b)>\sum_{j=1}^{n}\left(w_{j}|aR_{j}b+bQ_{j}a+bP_{j}a\right)$$

Na utilização do algoritmo S_2_, as alternativas têm sua atratividade pontuada em situações nas quais ocorre aP_j_b e aQ_j_b. Já na utilização do algoritmo S_3_, as alternativas têm sua atratividade pontuada em situações nas quais ocorre aP_j_b, aQ_j_b e aI_j_b. Nos algoritmos S_2_ e S_3_, verifica-se um cenário mais flexível, no qual uma menor diferença entre as alternativas permite classificar uma alternativa como melhor que a outra^24^.

Recomenda-se que o THOR seja utilizado preferencialmente em situações de pseudocritério e quase-critério, uma vez que o método pode ser utilizado em sua capacidade total. A utilização do THOR em situações de verdadeiro critério, quando os valores de p e q assumem valor igual a zero, leva à igualdade dos ordenamentos correspondentes a S_1_ e S_2_^20^^,^^22^.

Entre suas principais contribuições, destacam-se a aplicação do método multicritério THOR na reciclagem de resíduos no Brasil^20^ e em processos associados à saúde^22^. Gomes e Costa^18^ aplicaram esse método, juntamente com os métodos ELECTRE (I e II) e PROMETHÉE II, ao problema de escolha de modelos de pagamento eletrônico por cartão de crédito, e outros autores o utilizaram para estabelecer estratégias para compra de oportunidade de uma fragata para a Marinha brasileira^25^.

O THOR leva em consideração apenas a multiplicação pelo índice na situação aQ_j_b, deteriorando o ganho apenas nesse caso. O THOR 2 é uma evolução do método THOR original, que inclui também a depreciação da pontuação nas situações de preferência forte e indiferença. Dessa forma, o THOR 2 representa uma significativa contribuição, uma vez que toda a incerteza presente na atribuição das classificações das alternativas e dos pesos é quantificada.

Para viabilizar este estudo foi realizada pesquisa de literatura para obter os dados operativos e hospitalares de cada navio analisado. Por se tratar de navios militares, muitos dados são sigilosos, e portanto foram utilizados apenas parâmetros disponibilizados em fontes oficiais e ostensivas da Marinha do Brasil^26^^–^^29^.

Foram coletados também dados em artigos acadêmicos que abordam a atuação dos NAsH em ações cívico-sociais^9^^–^^11^. Essa pesquisa originou os dados a serem analisados (Tabela 1).

**Tabela 1:** Dados operativos e hospitalares dos navios avaliados.

Critério	Característica	NAsH Dr. Montenegro	NAsH Oswaldo Cruz	NAsH Soares Meirelles	NAsH Tenente Maximiano
	Comprimento (m)	42	47,2	63	31,06
	Largura	11	8,45	12	6,5
Manobrabilidade	Calado	2,4	1,75	2,1	1,02
	Descolamento máximo (ton)	347	490	1.338	160
Tripulação	Em número de pessoas	60	27	47	23
Raio de ação	Em milhas náuticas	3.200 (a 5 nós)	3.000 (a 7 nós)	6.000 (a 11 nós)	1.100 (a 11 nós)
Velocidade máxima descendo o rio	Em nós	10	12	12	12
Capacidade de evacuação de pacientes	Recursos de evacuação de pacientes	2 lanchas para transporte de pessoal	Convoo capaz de operar um helicóptero Bell Jet Ranger IH-6 ou Esquilo UH-12, além de 2 lanchas para transporte de pessoal	2 lanchas para transporte de pessoal	2 lanchas para transporte de pessoal
	Quantidade de leitos hospitalares disponíveis	6	6	6	3
Capacidade hospitalar	Atendimentos médicos disponíveis	3 consultórios, 2 gabinetes odontológicos, 1 laboratório, 1 farmácia, 1 sala de raio X, 2 enfermarias, 1 sala de cirurgia, 1 sala de emergência, UTI	2 ambulatórios, 2 gabinetes odontológicos, 1 laboratório, 1 farmácia, 1 sala de raio X, 2 enfermarias, 1 sala de cirurgia	Consultórios médicos, odontológicos, farmácia, sala de vacinação, sala de raio X, centro cirúrgico, enfermaria, laboratório de análises clínicas	Centro cirúrgico, enfermaria, sala de esterilização, sala de expurgo, farmácia, laboratório, consultório médico, consultórios odontológicos, 1 compartimento equipado com aparelho de raio-X

## RESULTADOS

A Tabela 2 apresenta alternativas, critérios, limiares de preferência, discordância e pesos dos critérios utilizados na análise. A primeira coluna é composta pelas alternativas, e as seis colunas seguintes, pelos critérios. Cada célula corresponde a uma alternativa de navio classificada no seu respectivo critério. As alternativas dos critérios manobrabilidade, capacidade hospitalar e evacuação de pacientes foram classificadas mediante uma escala de intervalos, na qual a diferença relativa entre os valores das alternativas foi levada em consideração.

**Tabela 2:** Alternativas, critérios, pesos e limiares de preferência e discordância.

Navios	Velocidade máxima	Tripulação	Raio de ação	Manobrabilidade	Capacidade hospitalar	Evacuação de pacientes
NAsH “Oswaldo Cruz”	10	-27	3.000	3	2	4
NAsH “Dr. Montenegro”	12	-60	3.200	3	2	2
NAsH “Soares Meirelles”	12	-47	6.000	1	3	2
NAsH “Tenente Maximiano”	12	-23	1.100	4	1	1

	Critérios	Peso atribuído	Valor de p	Valor de q	Discordância	
	Velocidade máxima	3	2	1	4,5	
	Tripulação	1	11	5	18	
	Raio de ação	2	1.000	300	2.500	
	Manobrabilidade	2	1	0,5	3,1	
	Capacidade hospitalar	4	1	0,5	2,9	
	Evacuação de pacientes	4	1	0,5	3,7	

Os valores de pesos, limites de preferência (p), limites de indiferença (q) e discordância para cada critério foram atribuídos por análise conjunta com especialistas da área.

Utilizando-se os dados apresentados na Tabela 2, foi possível gerar as ordenações relacionadas na Tabela 3. Os cálculos foram efetuados por meio de um sistema computacional denominado THOR 2, desenvolvido por Tenório et al.^30^ no Instituto Militar de Engenharia.

**Tabela 3:** Resultados obtidos após aplicação do método.

S_1_		S_2_		S_3_	
NAsH “Tenente Maximiano”	1,5	NAsH “Oswaldo Cruz”	1,881	NAsH “Oswaldo Cruz”	2,357
NAsH “Oswaldo Cruz”	1,5	NAsH “Soares Meirelles”	1,714	NAsH “Soares Meirelles”	1,857
NAsH “Dr. Montenegro”	1,5	NAsH “Tenente Maximiano”	1	NAsH “Tenente Maximiano”	1
NAsH “Soares Meirelles”	1,5	NAsH “Dr. Montenegro”	0,5	NAsH “Dr. Montenegro”	0,5

## DISCUSSÃO

Na situação S_1_, os quatro navios obtiveram exatamente a mesma pontuação, não sendo possível concluir qual seria o melhor navio para cumprir a missão.

Ao analisar o algoritmo S_2_, que considera as relações de sobreclassificação aP_j_b e aQ_j_b para ocorrer a dominância, observa-se que o NAsH “Oswaldo Cruz” apresenta soma ligeiramente superior ao “Soares Meirelles”, sendo matematicamente o melhor navio na análise proposta. Entretanto, na prática, os dois podem ser considerados tecnicamente empatados, pois a diferença relativa entre eles é extremamente pequena (0,167). Ainda no cenário S_2_, observa-se que os NAsH “Tenente Maximiano” e “Dr. Montenegro” apresentaram as menores notas, com diferenças relativas maiores em relação aos dois mais bem classificados, podendo ser descartados do processo de escolha.

Com relação ao algoritmo S_3_, a situação mais flexível, nota-se que o NAsH “Oswaldo Cruz” novamente obteve a maior soma, dessa vez com uma diferença relativa mais considerável para o segundo colocado, o que confirma sua escolha como o navio mais adequado a ser empregado no combate à pandemia.

Portanto, chega-se à seguinte classificação final: em primeiro lugar, NAsH “Oswaldo Cruz; em segundo, NAsH “Soares Meirelles”; em terceiro, NAsH “Tenente Maximiano”; e em quarto, NAsH “Dr. Montenegro”.

Avaliando os motivos que levaram à classificação final das alternativas, é possível observar que “Oswaldo Cruz” e “Soares Meirelles” obtiveram os dois melhores resultados porque apresentam as maiores capacidades de evacuação e hospitalar, respectivamente – os dois critérios com maior peso (4) atribuído pelos especialistas na análise proposta. Indo um pouco mais além, observa-se que o “Soares Meirelles” obtém a maior nota em três dos seis critérios avaliados, mas não apresenta boas notas nos restantes. Já o “Oswaldo Cruz” (o primeiro colocado) obtém a maior nota somente na capacidade de evacuação, porém mantém regularidade nos outros critérios, levando-o a ser o navio mais indicado. Talvez o fator primordial para a escolha do “Oswaldo Cruz” seja o fato de ser o único navio dentre as alternativas capaz de operar com aeronave, fazendo com que obtenha a maior nota da capacidade de evacuação e com maior distância relativa.

Em face do exposto, fica claro que o objetivo deste artigo foi atingido ao apontar o NAsH “Oswaldo Cruz” como o mais adequado para o combate à pandemia no Amazonas. Ressalta-se que o método THOR 2 pode ser utilizado para resolver problemas reais dos mais variados tipos – táticos, operacionais e estratégicos –, sendo assim um método de grande utilidade para a tomada de decisão.

Além disso, a facilidade, flexibilidade, confiabilidade e rapidez de aplicação do método podem facilitar sobremaneira os cálculos, muitas vezes complicados, que envolvem o apoio multicritério à decisão.

Por fim, sugere-se que este modelo de ordenação de alternativas usando o THOR 2 pode ser expandido a outras aplicações na área da saúde.
